# Severe recurrent hyponatremia in a 72-year-old patient with undiagnosed partially empty Sella syndrome—a case report

**DOI:** 10.1093/omcr/omad102

**Published:** 2023-09-25

**Authors:** Agnimshwor Dahal, Bhim Chauhan, Jeevan Gyawali, Astha Thapa, Prakash Dhungel, Saroj Yadav

**Affiliations:** Nepal Intensive Care Research Foundation, Kathmandu, Nepal; Patan Academy of Health Sciences, School of Medicine (PAHS-SOM), Lalitpur, Nepal; Patan Academy of Health Sciences, School of Medicine (PAHS-SOM), Lalitpur, Nepal; Nepal Intensive Care Research Foundation, Kathmandu, Nepal; Patan Academy of Health Sciences, School of Medicine (PAHS-SOM), Lalitpur, Nepal; Patan Academy of Health Sciences, School of Medicine (PAHS-SOM), Lalitpur, Nepal

## Abstract

Hyponatremia is one of the most common electrolyte abnormalities encountered in medical practice and is caused by multiple conditions. SIADH is the most common cause of hyponatremia, however, careful workup must be performed on all patients as mere supplementation may cause recurrent hyponatremia and serious side effects. Adrenal insufficiency is a principal culprit that mimics the clinical picture of SIADH and tends to worsen when treated in the line of SIADH. In addition, individuals may have various comorbidities, such as hypothyroidism in our case, which can also cause hyponatremia, making it difficult to determine precise etiology. We present a case of a 72-year-old man with recurrent hyponatremia, who was subsequently diagnosed as partially empty sella syndrome. Given the temporal relationship between the use of corticosteroids and the improvement of his symptoms, low cortisol and ACTH levels, adrenal insufficiency was most likely the cause of the hyponatremia in our patient.

## INTRODUCTION

Hyponatremia is the most prevalent electrolyte disturbance in hospitalized patients, with an incidence ranging from 5% to 30%, depending on the criteria, and associated with a high risk of morbidity and mortality [1]. Syndrome of inappropriate antidiuretic hormone secretion (SIADH) is the most common cause of hyponatremia, particularly in older patients with many comorbidities. It is a condition characterized by the excessive release of antidiuretic hormone (ADH) from the pituitary gland or other sources, leading to the retention of water by the kidneys and dilutional hyponatremia in the bloodstream. SIADH can be attributed to various factors like malignancies, medications, pulmonary diseases, surgery, etc. [2].

SIADH is characterized by a clinical picture that includes euvolemic hyponatremia, dilutional hypoosmolality, inappropriately concentrated urine, paradoxical natriuresis, and the absence of other causes of hyponatremia [2]. However, there are other possibilities to explore before reaching the diagnosis of SIADH, as there are other conditions that can have a similar clinical appearance. Adrenal insufficiency is the principal culprit that mimics the clinical picture of SIADH (hypoosmolar, euvolemic with paradoxical natriuresis) but tends to worsen when treated in the line of SIADH. Similarly, hypothyroidism also causes hyponatremia by mechanism similar to SIADH by releasing excessive ADH causing a similar laboratory picture as mentioned above [1]. As each of these causes requires a different approach to treatment, it is crucial to identify the actual cause of hyponatremia and treat accordingly.

A rare cause of secondary adrenal insufficiency, empty sella syndrome is a condition characterized by herniation of the subarachnoid space within the sella that’s usually accompanied by some flattening of the pituitary gland. Radiologically, the sella is defined as partially empty when less than 50% of it is filled with CSF and the pituitary gland thickness is ≥3 mm, or totally empty when more than 50% of the Sella is filled with CSF and the gland thickness is <2 mm in diameter [3]. Although uncommon, it is a potentially catastrophic form of secondary adrenal insufficiency. As cortisol is a physiological tonic inhibitor of ADH secretion, patients with adrenal insufficiency have unsuppressed release of ADH resulting in hyponatremia [4]. We discuss the case of a 72-year-old man with recurrent hyponatremia who was initially identified and treated as SIADH but was later diagnosed as partially empty sella syndrome (PESS) producing secondary adrenal insufficiency.

## CASE HISTORY

A 72 years male with known history of type II diabetes, hypertension, hypothyroidism, prostate cancer treated with chemotherapy and radiotherapy in the past and chronic radiation proctitis was presented by his family to the emergency department with complaints of altered mentation for a day associated with generalized body swelling starting from periorbital region for 3 days and generalized body weakness for a week.

Prior to this presentation, the patient had two similar episodes of decreased consciousness and abnormal mentation two weeks ago. Lab investigations then revealed hyponatremia (Serum sodium 118 mEq l^−1^ and 121 mEq l^−1^ respectively). The patient was given normal saline both times, which subsequently improved his mentation and he was returned home after attaining serum sodium levels of more than 125 mEq l^−1^.

6 months ago, the patient had presented to the outpatient department (OPD) with symptoms suggestive of hypothyroidism. Lab investigations confirmed the diagnosis of primary hypothyroidism and he was subsequently started on tablet thyroxine of 50 mcg daily. However, his symptoms had been persisting despite the medication, so a repeat thyroid profile test was sent two weeks ago. TFT revealed persistent hypothyroidism and as a result, the dosage of thyroid medication was escalated (50 + 25 + 12.5 mcg).

On examination in the emergency department (ED), he was neither oriented nor conscious with Glasgow Coma Scale (GCS) of 9/15 (E3 V2 M4). His vitals were within normal range (temperature: 98F, pulse: 80 beats per min, blood pressure: 116/78 mmHg, respiration rate: 16 breaths per min). There were no signs of focal weakness and deep tendon reflexes (DTR) were normal. Other systemic examinations including respiratory, cardiovascular, abdominal and musculoskeletal were unremarkable as well. The airway was protected, capillary blood glucose level was assessed (95 mg dl^−1^) and Foley catheterization was performed.

His laboratory investigations revealed severe hyponatremia (sodium: 113 mEq l^−1^; 135–145), hypokalemia (potassium: 2.9 mEq l^−1^; 3.5–5.5), and hypomagnesemia (magnesium: 0.8 mEq l^−1^; 1.7–2.2). Complete blood count analysis demonstrated low hemoglobin (8.5 mg dl^−1^; 13.5–17.5). However, other parameters were within normal limits (total count: 4900/mm^3^, 4000–11 000; Neutrophil 71 Lymphocyte 25, platelets: 180 000/mm^3^). A peripheral blood smear was done to look for the causes of anemia, revealing a normocytic hypochromic picture. Thyroid function test (TFT) showed elevated thyroid stimulating hormone (TSH) 22.97 μIU ml^−1^ (0.34–4.25) with normal free T4 1.04 ng dl^−1^ (0.7–1.24), and free T3 2.39 pg ml^−1^ (2.4–4.2) suggestive of subclinical hypothyroidism. Renal function (urea: 26 mg dl^−1^; 7–20), (creatinine: 1.1 mg dl^−1^; 0.6–1.3) and liver function (total bilirubin: 1.2 mg dl^−1^; 0.1–1.2), (direct bilirubin: 0.4 mg dl^−1^; 0–0.3), (aspartate transaminase: 34 U l^−1^; 10–40 U l^−1^), (alanine transaminase: 33 U l^−1^; 7–56), (alkaline phosphatase: 58 U l^−1^; 30–120), (total protein: 6.6 g dl^−1^; 6–8.3), (albumin: 4.3 g dl^−1^; 3.4–5.4) tests were, however, within normal limits. Viral serologies (HIV, hepatitis B, and C) were negative. Electrocardiogram findings were within normal limits.

He was then transferred to the intensive care unit (ICU). Hyponatremia was treated with controlled titration of 3% normal saline with the target of correcting sodium deficit by 6 mEq l^−1^ over 24 h. Hypokalemia and hypomagnesemia were treated with supplementation of potassium and magnesium respectively. Strict input and output charting was maintained. Workup for hyponatremia revealed low serum osmolality (276 mEq l^−1^; 280–295), high urine sodium (76 mEq l^−1^; 40–220), high urine osmolality (305.41 mOsm kg^−1^, 500–800) with normal urine specific gravity (1.015, 1.005–1.03). In addition, serum uric acid was low (3.3 mg dl^−1^; 3.4–7). As a result, he was diagnosed with SIADH and treatment was started. Arterial blood gas (ABG) evaluation was done to find his acid-base status which revealed mild metabolic acidosis (pH: 7.3, 7.35–7.45; pCO2: 25 mmHg, 35–45; HCO3: 20 mEq l^−1^, 22–28).

**Figure 1:**
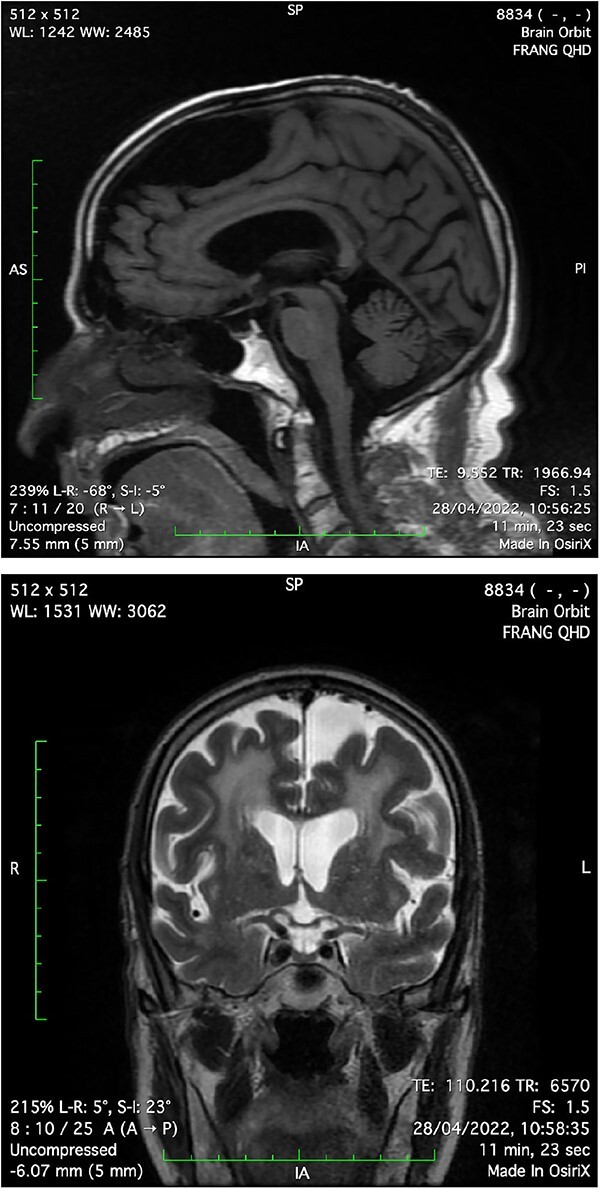
Sagittal T1 and coronal T2 sections of magnetic resonance imaging show marked thinning of the anterior lobe of the pituitary gland, about 1–2 mm in height in shallow sella turcica, and no evidence of suprasellar or para sellar mass.

As our patient had two similar episodes of altered mentation and hyponatremia within the last two weeks where he was treated with normal saline but hyponatremia recurred, We sent further investigations to determine the cause of recurrent hyponatremia despite treatment. Lab investigations revealed low cortisol on two separate occasions (3.17 and 3.6 ug dl^−1^, 6–23), low testosterone (0.49 ng ml^−1^, 1.75–1.81), and high prolactin (21.02 ng ml^−1^, 2.1–17.7) levels. On further investigation, early morning baseline plasma ACTH was also low, suggestive of secondary adrenal insufficiency. Evaluation of subclinical hypothyroidism further revealed low anti-thyroid peroxidase level (0.2 U ml^−1^, <9.0) suggestive of intact thyrotropes in the pituitary gland. Subsequently, a contrast-enhanced MRI was sent which revealed a partially empty sella (Fig. 1).

**Figure 2:**
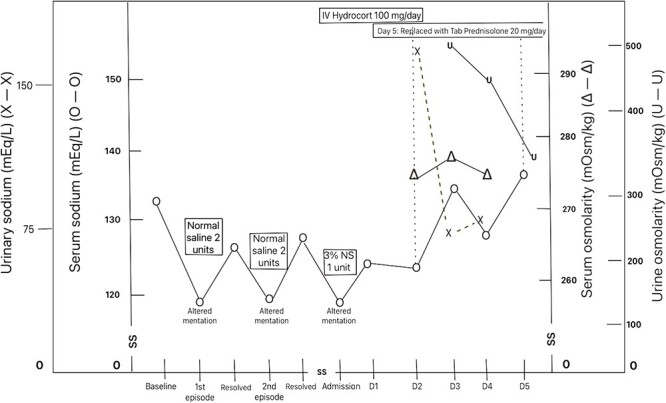
Trend of sodium level and osmolality of patient before and with treatment.

As a result, intravenous hydrocortisone was started for secondary adrenal insufficiency with which the patient’s clinical condition improved gradually as well as sodium level returned to normal over the course of ICU stay. Intravenous hydrocortisone was later switched to tablet prednisone and tablet thyroxine was continued in the previous dose (87.5 mcg per day). Patient was discharged after 5 days of ICU stay and on follow-up 1 week after discharge, he had no new complaints and his repeat serum sodium was normal (Fig. 2).

## DISCUSSION

Our patient is a 72-year-old man who presented to us with repeated episodes of severe symptomatic hyponatremia over the course of two weeks. Workup was done for hyponatremia, where he exhibited typical characteristics of SIADH, including hyponatremia in the presence of euvolemia, decreased serum osmolality, high urine osmolality (>100 mOsm kg^−1^) and paradoxical natriuresis (*U*na > 40 mMol l^−1^). Additionally, the low levels of serum uric acid and blood urea nitrogen further supported the diagnosis of SIADH, which is the most common cause of euvolemic hyponatremia [2]. However, when the hyponatremia was treated with normal saline and water restriction, he developed recurrent episodes causing multiple ED visits. Thus we further evaluated the patient to look for etiologies causing a similar laboratory picture of hyponatremia and discovered the patient had low serum ACTH, low cortisol, low testosterone and low PTH levels. Further evaluation with an MRI head with contrast revealed PESS. Herein, we discuss a case of secondary adrenal insufficiency as a result of PESS that can cause hyponatremia resembling SIADH that should be considered prior to treating patients.

Empty sella syndrome constitutes flattening of the pituitary gland to varying degrees resulting in various symptoms like headache, visual disturbances and features of hypopituitarism. It commonly occurs as a primary disorder or may less commonly be secondary due to a cerebral tumor, status post pituitary tumor removal, Sheehan’s syndrome etc. Hypopituitarism frequently occurs in empty sella syndrome, manifesting as either panhypopituitarism or isolated endocrine abnormalities such as secondary adrenal insufficiency, hypothyroidism, growth hormone deficiency, and hypogonadism. Secondary adrenal insufficiency occurs due to decreased secretion of adrenocorticotropic hormone (ACTH), leading to hypocortisolism [3]. In this condition, the physiological inhibition of antidiuretic hormone (ADH) by cortisol is absent, resulting in elevated ADH levels and subsequent hyponatremia [4]. In our case, the patient had secondary adrenal insufficiency due to hypopituitarism, confirmed by labs and MRI images, which was attributed to repeated episodes of hyponatremia. Similar findings have also been reported in other studies done previously. In one study, a 64-year-old woman treated for hyponatremia with hypertonic saline experienced recurrence due to primary empty Sella syndrome. [4] Pansare *et al*. reported a case of sudden hyponatremia in a 71-year-old with an ectopic pituitary gland [5]. Although SIADH commonly presents with a normal acid–base balance, there are instances where it can manifest as metabolic alkalosis. In contrast our patient had mild metabolic acidosis which is attributed to type 4 RTA as a result of ACTH deficiency [6].

In contrast to secondary adrenal insufficiency, hypothyroidism in our patient was due to a primary cause suggesting intact thyrotropes in the pituitary gland. Our patient had been diagnosed with primary hypothyroidism for the past 6 months, however, due to inadequate dose of thyroxine, he still had subclinical hypothyroidism. Hypothyroidism also causes euvolemic hyponatremia depicting a similar picture to SIADH. Warner *et al*. showed a statistical association between hyponatremia and hypothyroidism: for every 10 mU l^−1^ rise in thyroid-stimulating hormone, serum sodium decreased by 0.14 mmol l^−1^ [7]. However, we believe that primary hypothyroidism was not the underlying cause of the recurring episodes of hyponatremia in our case. While mild hyponatremia has been associated with hypothyroidism, thyroid hormone replacement and moderate fluid restriction are generally enough to correct serum sodium levels. Our patient had severe hyponatremia with altered mental status. Hypothyroidism should not be considered the sole cause of the low serum sodium in hypothyroid patients with TSH levels <50 mIU l^−1^ [8]. Our patient’s TSH was 22.97 μIU ml^−1^. Immediately after giving steroids and 3% NS, the patient’s condition improved and did not have further hyponatremic episodes on follow up with maintenance steroid dosage.

After coming to a proper diagnosis, our patient responded to glucocorticoid replacement and his symptoms along with hyponatremia gradually improved. During the current visit, his thyroxine dose was only adjusted after he responded to prednisolone with the principle hydrocortisone should be replaced before thyroxine to avoid worsening of hypocortisolism or even precipitating an adrenal crisis [9]. 15–20 mg hydrocortisone is recommended to replace in adrenal insufficiency [9]. One of the major principles in replacing corticosteroid treatment is to individualize treatment according to each patient. With the patient coming in with severe hyponatremia, altered mental status and the acuity of the condition, we decided to give a higher dose of hydrocortisone (100 mg/day in two divided doses) which was later changed to prednisone 20 mg. This was continued for 5 days and then maintained at appropriate dosage of prednisone 5 mg/day.

With the evidence of euvolemic hyponatremia, low baseline ACTH, low repeated cortisol, PTH and testosterone, partially empty sella on MRI, and the temporal relationship between administration of steroid and clinical improvement in our patient, we came to the conclusion that the cause of repeated hyponatremia was PESS secondary to hypopituitarism (hypocortisolism). Hence, we believe it is crucial to conduct a comprehensive diagnostic assessment to identify the specific cause while simultaneously correcting the sodium levels.

## Supplementary Material

ACTH_report_omad102

cortisol_omad102

## Data Availability

All data underlying the results are available as part of the article and no additional source data are required.
