# Supplementary material for: Severe recurrent hyponatremia in a 72-year-old patient with undiagnosed partially empty Sella syndrome—a case report
Source: Oxf Med Case Reports. 2023 Sep 25;2023(9):omad102. doi: 10.1093/omcr/omad102 (PMC10530318; doi:10.1093/omcr/omad102)
Supplement: ACTH_report_omad102 [file acth_report_omad102.pdf]

Crystal Diagnostic
Screening for Clarity

Crystal Diagnostic Lab
Sinamangal-9, Kathmandu

Phone: 014561020
Email: info@crystaldiagnosticlab.com
www.crystaldiagnosticlab.com

Sample ID:	**168618**		Sample ID: 168618
Patient's Name:	[illegible]		
Age:	73 yrs	Date Of Collection:	03 Mar 2022 07:32:12<br>2078/11/19 BS
Gender:	Male		
Referred By:	Self	Date Of Registration:	03 Mar2022 09:24:07<br>2078/11/19 BS
Requestor:	Nidan Hospital		
Contact No.:		Date Of Reporting:	03 Mar 2022 14:21:20<br>2078/11/19 BS
Email ID:	nidanpathology076@gmail.com		

Immunology/Serology

Test	Result	Reference	Unit
**ACTH, Plasma**	5	6 - 50	pg/ml

Reference range applies only to specimens collected between 7am-10am

*** End of Report ***

Sharwan Kumar Yadav
Hematologist (MSC. MLT)
21 Med. Haematology &
Blood Transfusion

Dr. Mahesh Subedi
Consultant Pathologist
MBBS,MD(Pathology) T.U.
NMC - 4596

Dr. Mukunda Sharma
Chief Consultant Pathologist
MBBS, DGO, MD(Pathology)
NMC-1859
