# Supplementary material for: Severe recurrent hyponatremia in a 72-year-old patient with undiagnosed partially empty Sella syndrome—a case report
Source: Oxf Med Case Reports. 2023 Sep 25;2023(9):omad102. doi: 10.1093/omcr/omad102 (PMC10530318; doi:10.1093/omcr/omad102)
Supplement: cortisol_omad102 [file cortisol_omad102.pdf]

Crystal Diagnostic
Screening for Clarity

Crystal Diagnostic Lab
Sinamangal-9, Kathmandu

Phone: 014561020
Email: info@crystaldiagnosticlab.com
www.crystaldiagnosticlab.com

Sample ID:	**169251**		
Patient's Name:	[illegible]		
Age:	73 yrs	Date Of Collection:	01 Mar 2022 14:58:37<br>2078/11/17 BS
Gender:	Male		
Referred By:	Self	Date Of Registration:	01 Mar 2022 14:58:37<br>2078/11/17 BS
Requestor:	Nidan Hospital		
Contact No.:		Date Of Reporting:	01 Mar 2022 17:13:43<br>2078/11/17 BS
Email ID:	nidanpathology076@gmail.com		

Sample ID: 169251

Immunology/Serology

Test	Result	Reference	Unit
**Testosterone (Total)**	0.49	Male: 1.75- 7.81<br>Female:<0.1-0.75	ng/ml

Testosterone is the main sex hormone (androgen) in men. It is generally produced in both male and female. However, testosterone levels are normally much higher in men than in women. This test is done to determine if the testosterone levels are abnormal, which may help to explain erectile dysfunction, infertility, or premature or delayed puberty in male, or masculine physical features, polycystic ovarian syndrome in female.

Test	Result	Reference	Unit
**Prolactin**	21.02	Male:2.1-17.7<br>Female:<br>Non pregnant:2.8-29.2<br>Pregnant:9.7-208.5<br>Post<br>menopausal:1.8-20.3	ng/ml

Prolactin is a hormone whose primary role is to promote breast milk production (lactation). It is normally elevated in women during pregnancy and just after childbirth. It is normally low in men and non-pregnant women.It helps to investigate unexplained flow of breast milk (galactorrhea), abnormal nipple discharge, absence of menstrual periods, and/or infertility in women; in men, to help diagnose the cause of decreased libido and/or erectile dysfunction; to detect and monitor a pituitary tumor that produces prolactin (prolactinoma).

Test	Result	Reference	Unit
**Cortisol**	3.17	Nomal Adult : 5.2 -35.0<br>Morning 7.26 - 32.28<br>Evening 3.24 - 15.0<br>Patient treated with<br>ACTH<br>28.0 - 60.0<br>Patient treated with<br>Dexamethasone < 5.0	µg/dl

Morning sample was received.

Test	Result	Reference	Unit
**Adrenocorticotropic Hormone (ACTH)**	**53.0**	6-48	pg/mL

Suraj Gautam	Dr. Mahesh Subedi	Dr. Mukunda Sharma
Sr. Medical Technologist	Consultant Pathologist	Chief Consultant Pathologist
A-202	MBBS,MD(Pathology) T.U.	MBBS, DGO, MD(Pathology)
	NMC - 4596	NMC-1859

Sample ID: 169251

Sample ID:	**169251**		
Patient's Name:	**Dr. Shyam Sundar Mishra**		
Age:	73 yrs	Date Of Collection:	01 Mar 2022 14:58:37<br>2078/11/17 BS
Gender:	Male		
Referred By:	Self	Date Of Registration:	01 Mar 2022 14:58:37<br>2078/11/17 BS
Requestor:	Nidan Hospital		
Contact No.:		Date Of Reporting:	01 Mar 2022 17:13:43<br>2078/11/17 BS
Email ID:	nidanpathology076@gmail.com		

*** End of Report ***

Suraj Gautam
Sr. Medical Technologist
A-202

Dr. Mahesh Subedi
Consultant Pathologist
MBBS,MD(Pathology) T.U.
NMC - 4596

Dr. Mukunda Sharma
Chief Consultant Pathologist
MBBS, DGO, MD(Pathology)
NMC-1859
